# Partial and full arc with DynamicARC technique in pencil beam scanning proton therapy for bilateral head and neck cancer: A feasibility and dosimetric study

**DOI:** 10.1002/acm2.14611

**Published:** 2024-12-20

**Authors:** Suresh Rana, Noufal Manthala Padannayil, Shyam Pokharel, Hina Saeed, Michael Kasper

**Affiliations:** ^1^ Department of Radiation Oncology, Lynn Cancer Institute, Boca Raton Regional Hospital Baptist Health South Florida Boca Raton Florida USA

**Keywords:** DynamicARC, head and neck cancer, pencil beam scanning, proton arc, proton therapy

## Abstract

**Purpose:**

A novel proton beam delivery method known as DynamicARC spot scanning has been introduced. The current study aims to determine whether the partial proton arc technique, in conjunction with DynamicARC pencil beam scanning (PBS), can meet clinical acceptance criteria for bilateral head and neck cancer (HNC) and provide an alternative to full proton arc and traditional intensity‐modulated proton therapy (IMPT).

**Method:**

The study retrospectively included anonymized CT datasets from ten patients with bilateral HNC, all of whom had previously received photon treatment. The clinical target volumes (CTV) were categorized into three levels: CTV_7000, CTV_5950, and CTV_5600. IMPT plans included three beams, whereas DynamicARC plans included dual‐partial‐arcs (DPA), single‐partial‐arc (SPA), and single‐full‐arc (SFA). All plans underwent robust optimization considering setup (± 3 mm) and range (± 3%) uncertainties applied to the CTVs. DynamicARC plans were evaluated against the NRG‐HN009 criteria and IMPT plans using various metrics.

**Results:**

All four techniques—IMPT, DPA, SPA, and SFA—demonstrated substantial compliance with NRG‐HN009 dosimetric criteria. DynamicARC produced superior dose conformity, lower hotspot, and improved homogeneity for high‐risk CTV compared to IMPT, with comparable performance for intermediate‐ and low‐risk CTVs. DynamicARC reduced the D_mean_ to the parotid glands by average differences of 14.5%–22.1% and to the oral cavity by an average difference of 15.75% compared to IMPT. DPA and SPA techniques achieved reductions in total integral dose of 3.7%–5.7% relative to IMPT. Overall, DPA yielded dosimetric results comparable to those of SFA while offering more conformal dose distributions and slightly better organ at risk sparing than SPA.

**Conclusion:**

On the ProteusOne with a partial gantry system, DPA and SPA, in conjunction with DynamicARC PBS protons, provided clear dosimetric advantages over three‐field IMPT. Future clinical implementation and further research into optimizing DynamicARC protocols are warranted to fully realize the benefits of these techniques in clinical settings.

## 1 INTRODUCTION

Recent advancements in proton therapy technology have led to the development of proton arc therapy (PAT).^1^ It offers the potential for conformal dose distributions and reduced toxicity compared to traditional proton therapy techniques such as intensity‐modulated proton therapy (IMPT) and uniform scanning proton therapy. Unlike IMPT, which targets the tumor from a few fixed angles,^2^ PAT irradiates the tumor from a multitude of angles,^1^, ^3^ employing an arc of proton beams to minimize doses to organs at risk (OARs).^3^, ^4^, ^5^, ^6^, ^7^

Several studies^5^, ^6^, ^8^, ^9^, ^10^, ^11^ have explored the dosimetric benefits of PAT for head and neck cancer (HNC). For instance, the study by Liu et al.^6^ investigated the feasibility and dosimetric improvements of PAT compared to IMPT for bilateral HNC. Their results demonstrated a reduction in mean doses to critical OARs, such as the ipsilateral and contralateral parotid glands and the oral cavity, by approximately 20−25%. de Jong et al.^5^ conducted a study that confirmed the dosimetric benefits of PAT in reducing toxicity for oropharyngeal cancer patients. Further reinforcing these dosimetric benefits, de Jong et al.^9^ expanded on the role of PAT within a model‐based clinic setting. Their study^9^ revealed that PAT not only outperformed IMPT in reducing integral doses and normal tissue complication probability (NTCP) values but also increased the number of patients qualifying for proton therapy.

However, the aforementioned studies^5^, ^6^, ^8^, ^9^, ^10^, ^11^ on PAT for HNC predominantly employed a full 360‐degree gantry and compared their results to those of IMPT. Specifically, Liu et al.,^6^ de Jong et al.,^5^, ^9^ Amstutz et al.,^8^ and Tattenberg et al.^11^ focused on the full arc gantry in their investigations, demonstrating the feasibility and dosimetric superiority of PAT within this context. Recently, the development of DynamicARC on the IBA ProteusOne machine (Ion Beam Applications, Louvain‐la‐Neuve, Belgium), featuring a partial gantry of 220 degrees, allows continuous irradiation and energy layer switching during gantry rotation.^1^, ^11^ With the increasing adoption of compact room proton therapy systems, which offer a more cost‐effective alternative to full gantry systems, it is essential to explore the feasibility and benefits of partial gantry PAT.

The current study focuses on evaluating the feasibility of employing the DynamicARC technique on an IBA ProteusOne PBS proton machine for treating bilateral HNC. Traditionally, IMPT for bilateral HNC involves the use of three to five static beams, with the need for couch kicks varying based on institutional protocols.^12^, ^13^ The novelty of the current study lies in its exploration of partial proton arcs in bilateral HNC treatment. Thus, the current study aims to determine whether the partial proton arc technique, in conjunction with the DynamicARC pencil beam scanning (PBS), can meet clinical acceptance criteria for bilateral HNC and provide a viable alternative to single full arc and traditional IMPT.

## 2 METHODS

### 2.1 Target volumes, OARs, and proton beam model

This Institutional Review Board (IRB)‐approved retrospective treatment planning study included anonymized computed tomography (CT) datasets of ten bilateral head and neck (diagnosis: oropharyngeal cancer) patients who had previously undergone photon therapy. The clinical target volumes (CTV) were categorized into three levels: CTV_7000, CTV_5950, and CTV_5600. The mean volumes for CTV_7000, CTV_5950, and CTV_5600 were 120.9 ± 40.8 cc, 281.9 ± 98.2 cc, and 206.9 ± 66.9 cc, respectively. Among these ten patients, the high‐risk CTV_7000 was located on the right side in 6 patients and on the left side in 4 patients. Treatment plans were developed in the RayStation treatment planning system (research version 2023B; RaySearch Laboratories, Stockholm, Sweden) and utilized the beam model based on the IBA ProteusOne PBS proton machine. The in‐air spot size (1σ) at the isocenter ranged from 3.4 mm at 225 MeV to 7.6 mm at 70 MeV. All plans in the current study assumed a constant relative biological effectiveness (cRBE) of 1.1.

### 2.2 IMPT planning

**FIGURE 1:**
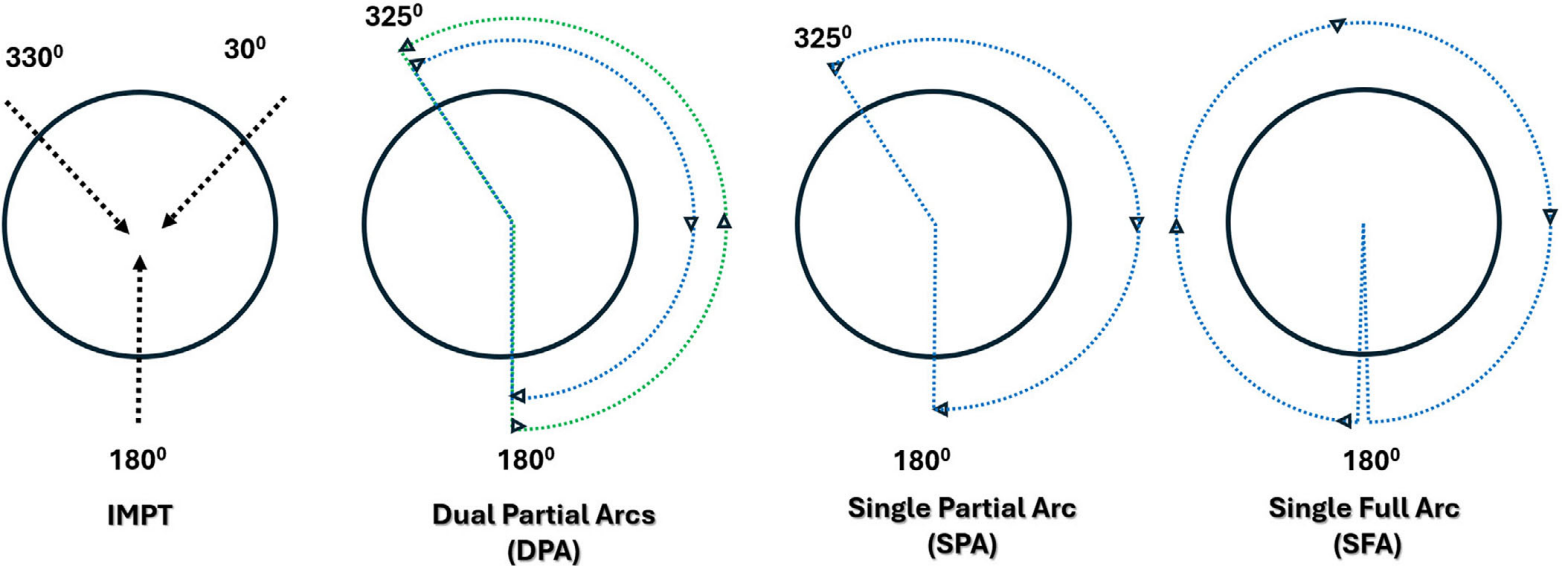
Static 3‐field IMPT and arcs configuration in DynamicARC PBS proton therapy for bilateral HNC treatment, with a couch angle of 0° for all setups. The 3‐field IMPT approach includes beam angles at 330°, 30°, and 180°. DynamicARC techniques consist of DPA covering clockwise (325° to 180°) and counterclockwise (180° to 325°) directions, SPA from 325° to 180° in a clockwise direction, and SFA spanning from 181° to 180° in a clockwise direction. DPA, dual‐partial‐arcs; HNC, head and neck cancer; IMPT, intensity‐modulated proton therapy; PBS, pencil beam scanning; SPA, single‐partial‐arc; SFA, single‐full‐arc.

The three‐beam IMPT technique was employed for all ten patients by including right‐anterior oblique (RAO) (gantry angle: 330°; couch angle 0°), left‐anterior oblique (LAO) (gantry angle: 30°; couch angle 0°), and posterior‐anterior beam (PA) (gantry angle: 180°; couch angle 0°). [Figure 1] We utilized a range shifter of 4 cm water equivalent thickness (WET) for all three beams. Several optimization structures were developed before the plan optimization process to guide and control spot placement within the target volumes. Spot assignment structures (SAS) and a PA block structure were utilized to facilitate the spot placement. The RAO‐SAS ensured that proton spots covered only the RAO area, while the LAO‐SAS similarly constrained spot placement to the LAO region. Spot placement for the PA beam was restricted to regions above the shoulder, ensuring that this beam treated only the upper target volumes. In the lower anterior neck regions, spot placement was strategically assigned to the RAO and LAO beams, ensuring appropriate coverage while minimizing exposure to the OARs. To prevent excessive dose spillage in the posterior neck from the PA beam, a PA spot block was optimized to avoid direct spot placement through the spinal cord, thereby reducing unnecessary dose to the posterior neck. The energy layer and spot spacing were set to automatic with a scale of 1.

A nominal IMPT treatment plan was generated using a multi‐field optimization (MFO) technique in conjunction with the Monte Carlo algorithm (10 000 ions/spot) and robust optimization (accounting for systematic setup error: ± 3 mm; range error: ± 3%). Based on the specified setup and range uncertainties, RayStation optimized each IMPT plan across 21 scenarios to deliver doses to the following target volumes: CTV_7000 treated for 70 Gy(RBE), CTV_5950 treated for 59.5 Gy(RBE), and CTV_5600 treated for 56 Gy(RBE), in 35 fractions using a simultaneous integrated boost (SIB) technique. The robust optimization objective was applied exclusively to the CTVs for parameters such as minimum dose, maximum dose, and minimum DVH. For serial OARs, maximum dose constraints were optimized, while parallel OARs were optimized using equivalent uniform dose (EUD) objectives. Plan optimization continued until all clinical goals were achieved, except for the submandibular glands, which overlapped with the target volumes. The final dose calculations were performed using the Monte Carlo algorithm (0.5% statistical uncertainty) with a 3 mm grid size. All IMPT plans were normalized such that the dose to 98% of CTV_7000 (D_98%_) received 70 Gy(RBE).

### 2.3 DynamicARC

#### 2.3.1 DynamicARC optimization

Engwall et al.^3^ introduced the Early Layer and Spot Assignment (ELSA) algorithm for optimization, designed to enable faster delivery in proton PBS arc therapy. The algorithm follows several key steps, starting with the definition of energy layers based on the patient's geometry and the gantry's start and stop angles.^3^ The arc is then divided into sectors, where one energy layer is selected per direction within each sector.^3^ It ensures that energy levels decrease across the sector, with upward energy jumps only allowed between sectors.^3^ The selected energy layers are evaluated for target coverage, with an optional penalty for upward jumps, and the optimal set of layers is used for spot weight optimization.^3^ By restricting the number of energy layers per direction to one, ELSA facilitates continuous delivery and minimizes energy layer switching.^3^ This reduces treatment time while preserving highplan quality. Robust optimization in DynamicARC is achieved using the worst‐case robust optimization method proposed by Fredriksson et al.^14^ By sequencing energy layers in descending order and limiting upward jumps, the method reduces switching time—critical for avoiding delays caused by transitioning between higher and lower energies during treatment delivery.^3^ For a comprehensive understanding of the robust optimization of DynamicARC, readers are encouraged to consult the publication by Engwall et al.^3^

#### 2.3.2 DynamicARC planning

For DynamicARC planning in the current study, no range shifters were utilized because previous studies^6^, ^10^ demonstrated that with the increased optimization freedom and multiple beam directions available in proton arc planning, the range shifter becomes unnecessary for bilateral HNC. We used a gantry angle spacing of 2° with a gantry angle window set to 1°. The gantry angle window is defined by a start and stop gantry angle.^3^ The energy layer spacing was set to automatic with a scale of 0.25, whereas the spot spacing was kept constant at 0.5 cm. Unlike IMPT, the DynamicARC plan did not use SAS. Instead, new avoidance blocks were generated to prevent the proton beam from passing through the shoulder regions, lungs, and heart. Both IMPT and DynamicARC plans shared the same optimization objectives for the CTVs and OARs and employed identical robust optimization settings, ensuring consistency in achieving clinical goals.

For each patient, three sets of DynamicARC plans were generated. First, a dual partial arcs (hereafter referred to as DPA) plan was robustly optimized. In the DPA plan, the arcs included one clockwise (gantry angles: 325⁰→180⁰; couch 0⁰) and one counterclockwise (gantry angles: 180⁰→325⁰; couch 0⁰). [Figure 1] Optimization continued for DPA plans until the desired clinical goals were met. Second, a single partial arc (hereafter referred to as SPA) plan was generated based on the DPA plan but with the deletion of a counterclockwise arc. Third, a single full arc (SFA) plan was generated based on a SPA plan but with a gantry rotation from 181⁰ to 180⁰. For a given technique, gantry angles and arc direction were consistent across all patients rather than being adjusted based on the laterality of CTV_7000. The final dose calculations were performed using the Monte Carlo algorithm (statistical uncertainty = 0.5%; grid size = 3 mm). All DynamicARC plans (DPA, SPA, and SFA) were normalized such that D_98%_ to the CTV_7000 is 70.0 Gy(RBE) per NRG HN009.

### 2.4 Plan quality

Both IMPT and DynamicARC plans were normalized such that 98% of CTV_7000 received 70 Gy(RBE). Treatment plans were evaluated based on the NRG HN009 dosimetric acceptance criteria (Table 1). Additionally, conformity index (CI), homogeneity index (HI), and integral dose (ID) were calculated.

(1) $$CI=\frac{TVDp}{TV}\times \frac{TVDp}{VDp}$$

where TVD_p_, TV, and VD_p_ are the target volume covered by the prescribed dose, target volume, and the volume enclosed by the prescription isodose line, respectively.

(2) $$HI=\frac{D_{5}}{D_{95}}$$

where D_5_ and D_95_ are the dose covering 5% and 95% of the volume of a structure, respectively.

(3) $$ID=\bar{D}.V$$

where $\bar{D}$ (Gy) is the mean dose delivered to volume V (L) (where L—liter).^15^

**TABLE 1:** AVERAGE ± STDEV results in IMPT and DynamicARC PBS proton plans (DPA, SPA, and SFA) of bilateral HNC. For each metric of given structure, the results are averaged over ten analyzed patients.

Structure	Metric	NRG‐HN009	IMPT	DPA	*p*‐value (DPA vs. IMPT)	SPA	*p*‐value (SPA vs. IMPT)	SFA	*p*‐value (SFA vs. IMPT)
CTV_7000	D_98%_ [Gy(RBE)]	70	70.0	70.0	N/A	70.0	N/A	70.0	N/A
CTV_7000	D_99%_ [Gy(RBE)]	≥66.5	69.7 ± 0.1	69.8 ± 0.1	0.106	69.7 ± 0.1	1.000	69.8 ± 0.1	0.344
CTV_7000	D_0.03cc_ [Gy(RBE)]	≤77	76.7 ± 1.1	75.2 ± 0.6	0.002	76.2 ± 0.8	0.344	75.9 ± 1.0	0.002
CTV_7000	D_95%_ [Gy(RBE)] WCS	≥70^*^	69.8 ± 0.3	69.7 ± 0.3	0.754	69.2 ± 0.4	0.022	69.5 ± 0.3	0.289
CTV_7000	CI		0.64 ± 0.04	0.69 ± 0.04	0.002	0.67 ± 0.04	0.022	0.68 ± 0.04	0.002
CTV_7000	HI		1.05 ± 0.01	1.04 ± 0.00	0.022	1.05 ± 0.00	1.000	1.04 ± 0.00	0.109
CTV_5950	D_98%_ [Gy(RBE)]	≥59.5	61.5 ± 1.2	61.1 ± 1.3	0.049	61.2 ± 1.2	0.109	61.1 ± 1.4	0.109
CTV_5950	D_95%_ [Gy(RBE)] WCS	≥59.5	61.3 ± 1.4	60.8 ± 1.5	0.002	60.5 ± 1.4	0.004	60.5 ± 1.4	0.008
CTV_5600	D_98%_ [Gy(RBE)]	≥56	56.6 ± 0.2	56.6 ± 0.3	0.922	56.6 ± 0.3	0.754	56.6 ± 0.2	0.754
CTV_5600	D_95%_ [Gy(RBE)] WCS	≥56	56.4 ± 0.3	56.3 ± 0.2	0.169	55.6 ± 0.5	0.109	55.8 ± 0.5	0.289
Brachial Plexus (Contralateral)	D_0.03cc_ [Gy(RBE)]	≤66	62.6 ± 2.1	61.7 ± 2.1	0.275	62.3 ± 1.6	0.921	61.5 ± 1.8	0.052
Brachial Plexus (Ipsilateral)	D_0.03cc_ [Gy(RBE)]	≤66	66.9 ± 4.0	65.4 ± 2.9	0.049	66.1 ± 3.3	0.232	65.2 ± 3.4	0.019
Brainstem PRV03	D_0.03cc_ [Gy(RBE)]	≤52	36.7 ± 3.2	14.9 ± 1.9	0.002	15.2 ± 2.3	0.004	14.8 ± 1.5	0.004
Cochlea (Contralateral)	D_Mean_ [Gy(RBE)]	≤30	17.7 ± 14.9	0.8 ± 0.7	0.004	0.8 ± 0.7	0.004	1.0 ± 1.0	0.012
Cochlea (Ipsilateral)	D_Mean_ [Gy(RBE)]	≤30	27.5 ± 12.8	1.3 ± 1.0	0.002	1.2 ± 1.0	0.002	1.5 ± 1.0	0.002
Esophagus	D_Mean_ [Gy(RBE)]	≤30	10.0 ± 7.2	9.6 ± 7.3	0.193	9.4 ± 7.2	0.064	9.8 ± 7.3	0.432
External	ID [Gy(RBE) ‐L]		138.7 ± 28.2	133.5 ± 27.5	0.025	130.7 ± 26.8	0.002	138.3 ± 29.5	0.846
External	V_3Gy_ [cc]		4219.6 ± 784.4	4928.7 ± 906.4	0.002	4717.8 ± 908.2	0.0039	5513.8 ± 1086.9	0.002
External‐PTV^b^	D_1cc_ [Gy(RBE)]	≤73.5	72.8 ± 1.1	71.0 ± 0.7	0.002	72.3 ± 1.4	0.1484	71.5 ± 0.9	0.008
Eye (Contralateral)	D_0.03cc_ [Gy(RBE)]	≤55	0.5 ± 0.8	0.7 ± 0.9	0.105	0.2 ± 0.4	0.002	0.4 ± 0.9	0.084
Eye (Ipsilateral)	D_0.03cc_ [Gy(RBE)]	≤55	0.5 ± 0.6	0.6 ± 1.2	0.625	0.5 ± 1.6	0.084	0.9 ± 2.6	0.105
Larynx^a^	D_Mean_ [Gy(RBE)]	≤30	23.4 ± 6.2	14.1 ± 4.0	0.002	14.3 ± 4.0	0.002	14.2 ± 3.9	0.002
Lens (Contralateral)	D_0.03cc_ [Gy(RBE)]	≤15	0.4 ± 0.7	0.2 ± 0.6	0.002	0.1 ± 0.2	0.002	0.1 ± 0.2	0.014
Lens (Ipsilateral)	D_0.03cc_ [Gy(RBE)]	≤15	0.4 ± 0.6	0.2 ± 0.7	0.014	0.2 ± 0.7	0.002	0.1 ± 0.4	0.002
Lips	D_Mean_ [Gy(RBE)]	≤20	5.9 ± 1.9	5.6 ± 1.9	0.625	5.2 ± 1.7	0.322	4.3 ± 1.4	0.037
Temporal Lobe (Contralateral)	D_0.03cc_ [Gy(RBE)]	≤70	13.8 ± 13.9	7.3 ± 9.2	0.160	6.8 ± 7.4	0.084	8.2 ± 9.9	0.131
Temporal Lobe (Ipsilateral)	D_0.03cc_ [Gy(RBE)]	≤70	20.4 ± 10.9	11.4 ± 11.6	0.002	9.2 ± 11.2	0.004	9.6 ± 10.0	0.002
Mandible	D_0.03cc_ [Gy(RBE)]	≤73.5	70.0 ± 5.0	69.5 ± 4.9	0.160	70.2 ± 4.8	0.846	70.0 ± 4.8	0.846
Optic Chiasm	D_0.03cc_ [Gy(RBE)]	≤54	0.4 ± 0.1	0.1 ± 0.1	0.002	0.1 ± 0.0	0.002	0.1 ± 0.0	0.002
Optic Nerve (Contralateral)	D_0.03cc_ [Gy(RBE)]	≤54	0.3 ± 0.2	0.2 ± 0.4	0.084	0.1 ± 0.1	0.002	0.1 ± 0.1	0.002
Optic Nerve (Ipsilateral)	D_0.03cc_ [Gy(RBE)]	≤54	0.3 ± 0.3	0.2 ± 0.5	0.084	0.1 ± 0.2	0.002	0.2 ± 0.5	0.105
Oral Cavity^a^	D_Mean_ [Gy(RBE)]	≤30	19.7 ± 3.8	16.6 ± 2.6	0.010	16.6 ± 2.7	0.010	16.7 ± 2.7	0.010
Parotid (Contralateral)	D_Mean_ [Gy(RBE)]	≤26	20.3 ± 3.9	16.0 ± 4.1	0.002	15.9 ± 4.1	0.002	16.0 ± 4.2	0.002
Parotid (Ipsilateral)	D_Mean_ [Gy(RBE)]	≤26	26.8 ± 5.4	22.1 ± 5.7	0.002	22.5 ± 6.0	0.002	22.4 ± 5.9	0.002
Pharynx^a^	D_Mean_ [Gy(RBE)]	≤45	34.9 ± 5.4	27.8 ± 4.2	0.002	27.9 ± 4.2	0.002	27.7 ± 4.2	0.002
Spinal Cord	D_0.03cc_ [Gy(RBE)]	≤45	25.7 ± 3.7	10.1 ± 0.5	0.002	10.7 ± 0.4	0.002	10.2 ± 0.3	0.002
Spinal Cord PRV05	D_0.03cc_ [Gy(RBE)]	≤50	36.7 ± 3.9	18.2 ± 2.4	0.002	18.8 ± 3.1	0.004	17.7 ± 2.1	0.004
Submandibular (Contralateral)	D_Mean_ [Gy(RBE)]	≤39	50.0 ± 9.4	48.9 ± 8.9	0.084	49.6 ± 8.9	0.375	49.0 ± 8.9	0.323
Submandibular (Ipsilateral)	D_Mean_ [Gy(RBE)]	≤39	64.2 ± 7.2	63.7 ± 7.1	0.064	64.0 ± 7.1	0.432	63.8 ± 7.1	0.064
Thyroid	D_Mean_ [Gy(RBE)]	≤50	49.1 ± 4.2	46.9 ± 4.8	0.002	47.4 ± 4.8	0.004	47.1 ± 4.6	0.002

*Note*: ^a^means target volume is subtracted if it overlaps with the OAR. ^b^PTV (3 mm uniform expansion around the CTV) is generated for the reporting purpose only. * means CTV_7000 D_95%_ WCS ≥ 68.6 Gy(RBE) is acceptable.

Abbreviations: CTV, clinical target volumes; DPA, dual partial arc; HNC, head and neck cancer; IMPT, intensity‐modulated proton therapy; PBS, pencil beam scanning; SPA, single partial arc; SFA, single full arc.

### 2.5 Plan robustness

Plan robustness was assessed under conditions of ± 3% range uncertainty and ± 3 mm setup uncertainty. Specifically, range and setup uncertainties were combined to generate 12 distinct scenarios. The worst‐case scenario at D_95%_ (D_95%_WCS_) was then calculated for CTV_7000, CTV_5950, and CTV_5600.

### 2.6 Beam delivery time

A simple time model was employed to determine the delivery time of DPA, SPA, and SFA plans. The time model has been described by Engwall et al.^3^ in which the spot irradiation time follows the relationship given in Pfeiler et al.^16^ In the current study, we used the mechanical parameters for ProteusOne: maximum gantry speed = 6° s^−1^, maximum acceleration = 0.6° s^−2^, upward energy layer switching time = 6 s, downward energy layer switching time = 0.8 s, dead time between energy layers = 0.3 s, and switching time between two spots = 2 ms.

### 2.7 Dosimetric data analysis

The differences in dosimetric parameters across various treatment plans were assessed. The initial phase of the analysis involved a comparative evaluation of the IMPT and DynamicARC plans against the NRG HN009 dose constraint guidelines. In the subsequent phase, each DynamicARC plan—namely, the DPA, SPA, and SFA—was individually compared to the IMPT plan, which served as the reference standard for this study. For statistical analysis, dosimetric results from both the IMPT and DynamicARC (DPA, SPA, and SFA) plans were analyzed using a paired, two‐tailed nonparametric Wilcoxon signed‐rank test. A *p*‐value of less than 0.05 was considered to indicate statistical significance.

## 3 RESULTS

### 3.1 CTVs

**FIGURE 2:**
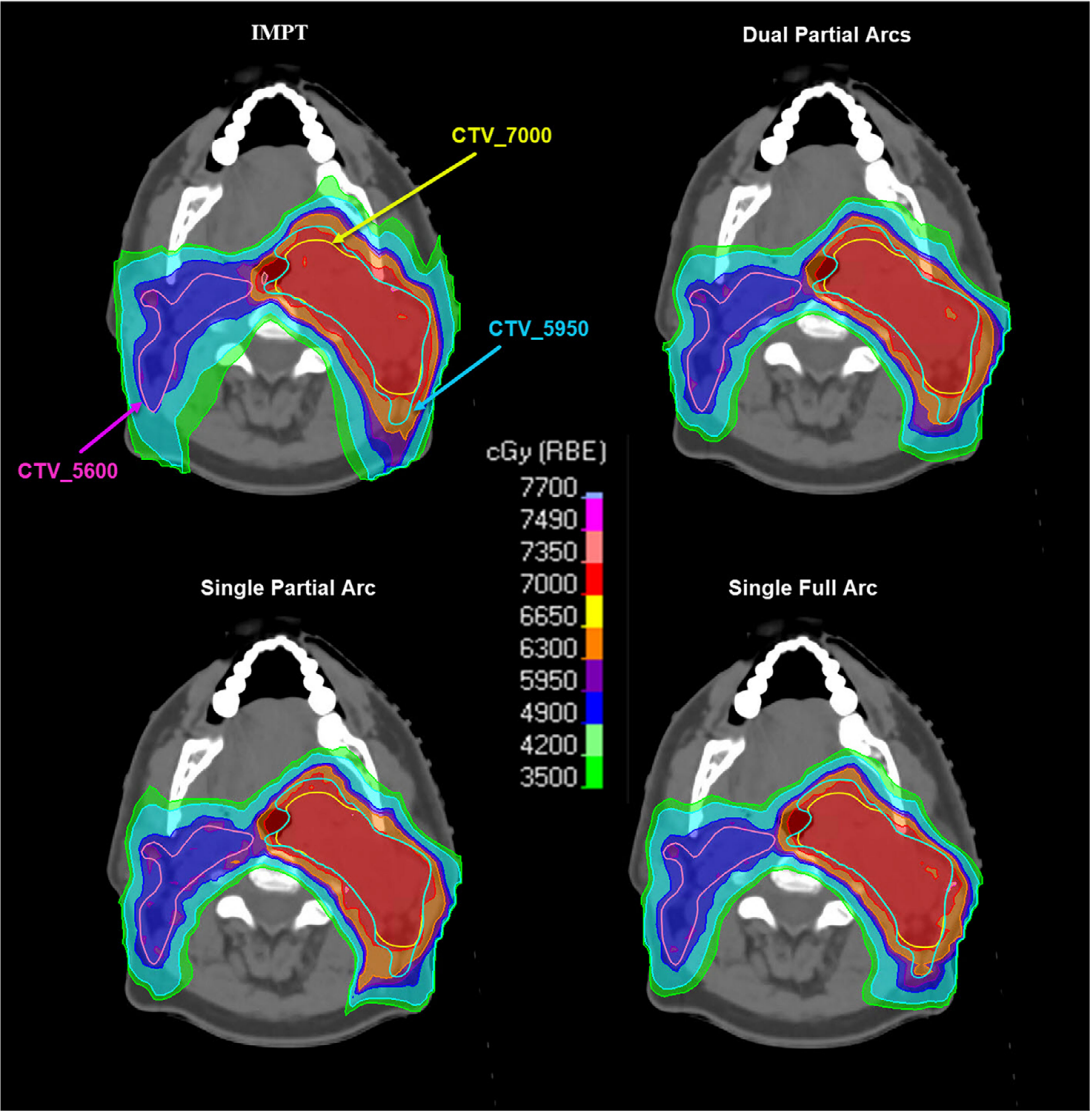
Dose distributions in 3‐field IMPT and DynamicARC PBS proton therapy plans of an example bilateral HNC patient. DynamicARC PBS proton plans are generated using DPA, SPA, and SFA techniques. DynamicARC plans in this figure demonstrate improved dose conformality compared to IMPT plan. DPA, dual‐partial‐arcs; HNC, head and neck cancer; IMPT, intensity‐modulated proton therapy; PBS, pencil beam scanning; SPA, single‐partial‐arc; SFA, single‐full‐arc.

All the CTV results (mean ± stdev) presented in this section and Table 1 are averaged over ten analyzed patients. Figure 2 shows the dose distributions in IMPT, DPA, SPA, and SFA plans of an example patient.

#### 3.1.1 Target coverage

For the high‐risk CTV_7000, the NRG HN009 D_98%_ criterion of 70.0 Gy(RBE) was achieved by all techniques, including IMPT and the DynamicARC (DPA, SPA, and SFA), through plan normalization. The D_99%_ to CTV_7000 was consistent across techniques. For the medium‐risk CTV_5950 and the low‐risk CTV_5600, all techniques met the D_98%_ criterion. [Table 1]

#### 3.1.2 Hot spot, HI, and CI

For the hot spot (D_0.03cc_) in CTV_7000, IMPT delivered 76.7 ± 1.1 Gy(RBE), while DPA and SFA delivered lower values of 75.2 ± 0.6 Gy(RBE) and 75.9 ± 1.0 Gy(RBE), respectively. Both DPA and SFA demonstrated significantly lower D_0.03cc_ compared to IMPT (*p* = 0.002). No significant difference was observed between IMPT and SPA (76.2 ± 0.8 Gy(RBE), *p* = 0.344).

As shown in Table 1, the HI for IMPT was 1.05 ± 0.01, while DPA and SFA showed slightly better homogeneity, with values of 1.04 ± 0.00. A significant difference was found between IMPT and DPA (*p* = 0.022), though no significant differences were observed between IMPT and SPA or SFA (*p* > 0.05). In terms of CI, IMPT demonstrated a lower value of 0.64 ± 0.04 compared to DPA (0.69 ± 0.04), SPA (0.67 ± 0.04), and SFA (0.68 ± 0.04). The differences in CI between IMPT and DynamicARC were statistically significant (*p* < 0.05), indicating improved dose conformity with DynamicARC techniques.

#### 3.1.3 Plan robustness

**FIGURE 3:**
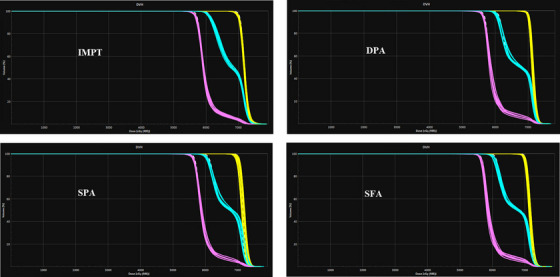
Robustness of CTV_7000 (yellow), CTV_5950 (blue), and CTV_5600 (pink) in IMPT and DynamicARC PBS proton therapy plans of an example bilateral HNC patient. DynamicARC PBS proton plans are generated using DPA, SPA, and SFA techniques. Each CTV has 12 perturbed scenarios, which were generated by combining ± 3% range uncertainty and ± 3 mm setup uncertainty. CTV, clinical target volumes; DPA, dual‐partial‐arcs; HNC, head and neck cancer; IMPT, intensity‐modulated proton therapy; PBS, pencil beam scanning; SPA, single‐partial‐arc; SFA, single‐full‐arc.

Figure 3 shows the plan robustness in IMPT, DPA, SPA, and SFA of an example patient. For D_95%_WCS_ CTV_7000, none of the techniques reached the preferred threshold of ≥70.0 Gy(RBE), with doses ranging from 69.2 ± 0.4 Gy(RBE) to 69.8 ± 0.3 Gy(RBE). However, all techniques met the acceptable limit of 68.6 Gy(RBE) for CTV_7000. No significant differences were observed between IMPT and DPA (*p* = 0.754) or SFA (*p* = 0.289), though SPA demonstrated a statistically significant difference compared to IMPT (*p* = 0.022). For D_95%_WCS_ to the CTV_5950, all techniques surpassed the preferred threshold of 59.5 Gy(RBE). Significant differences were noted between IMPT and DPA (*p* = 0.002), SPA (*p* = 0.004), and SFA (*p* = 0.008), indicating slightly higher D_95%_WCS_ to the CTV_5950 with IMPT compared to the DynamicARC techniques. In terms of D_95%_WCS_ to CTV_5600, all techniques exceeded the preferred threshold of 56.0 Gy(RBE), with no significant differences between IMPT and the DynamicARC techniques (*p* > 0.05).

### 3.2 OARS

**FIGURE 4:**
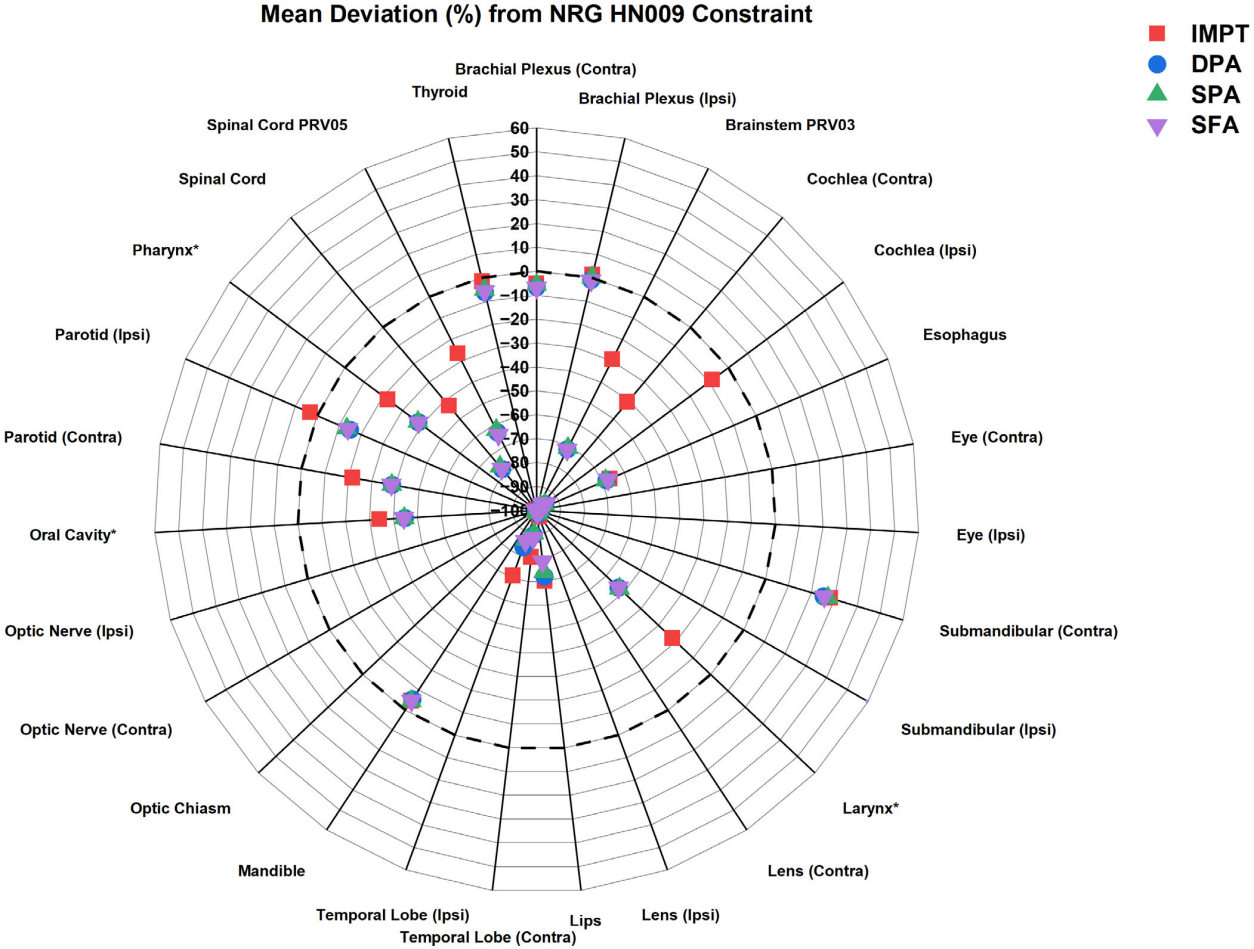
Mean deviation (%) in dosimetric metrics for IMPT and DynamicARC techniques (DPA, SPA, and SFA) from NRG HN009 dose constraints based on ten analyzed cases. Negative percentage differences indicate that the OAR result is below the NRG HN009 dose constraint and meets the compliance criteria, while positive differences indicate that it fails to meet the criteria. * means target volume is subtracted if it overlaps with the OAR. DPA, dual‐partial‐arcs; IMPT, intensity‐modulated proton therapy, OAR, organ at risk; SPA, single‐partial‐arc; SFA, single‐full‐arc.

All the OARs results (mean ± stdev) presented in this section and Table 1 are averaged over ten analyzed patients. The dosimetric evaluation of IMPT, DPA, SPA, and SFA, relative to NRG compliance criteria, revealed notable differences in OAR sparing. [Table 1 and Figure 4]

For the D_mean_ to the oral cavity, all DynamicARC techniques provided superior sparing compared to IMPT while maintaining compliance with NRG criteria. DPA demonstrated the most substantial D_mean_ reduction (−14.3 ± 14.4%, *p* = 0.010). SPA and SFA followed closely, with D_mean_ decreases of −13.9 ± 14.6% (*p* = 0.010) and −13.8 ± 14.3% (*p* = 0.010), respectively, when compared to IMPT. In the pharynx, all techniques remained compliant, and DPA and SFA showed the largest reductions in D_mean_ relative to IMPT (−20.3 ± 4.3%, *p* = 0.002 and −20.5 ± 4.2%, *p* = 0.002, respectively), providing the most effective pharynx sparing. SPA followed with a D_mean_ reduction of −20.0 ± 4.3% (*p* = 0.002) compared to IMPT.

Compliance was achieved across all DynamicARC techniques for both parotid glands. DPA consistently showed decreases in D_mean_ compared to IMPT, with reductions of −22.1 ± 8.3% (*p* = 0.002) for the contralateral parotid and −18.1 ± 4.4% (*p* = 0.002) for the ipsilateral parotid. SPA and SFA demonstrated similar reductions in D_mean_ relative to IMPT for the contralateral parotid (−21.9 ± 8.4% and −21.8 ± 8.7%, respectively; *p* = 0.025 for both) and ipsilateral parotid (−17.1 ± 5.3% and −17.5 ± 5.3%, *p* = 0.002 for both).

For D_mean_ to the larynx, esophagus, and thyroid, all DynamicARC techniques remained compliant with NRG criteria and outperformed IMPT. For the larynx, DPA provided the greatest reduction in D_mean_ compared to IMPT (−38.1 ± 13.4%, *p* = 0.002), followed by SFA (−38.0 ± 13.1%) and SPA (−37.4 ± 13.1%, *p* = 0.002). Reductions in the esophagus D_mean_ relative to IMPT were less pronounced. Specifically, DPA showed a modest decrease (−1.3 ± 32.9%, *p* = 0.193), SPA showed a slightly greater reduction (−4.9 ± 26.1%, *p* = 0.064), while SFA exhibited a small increase (1.2 ± 30.4%, *p* = 0.432). For the thyroid, all DynamicARC techniques achieved significant reductions in D_mean_ compared to IMPT, with DPA providing the largest reduction (−4.6 ± 3.3%, *p* = 0.002), followed by SFA (−4.2 ± 3.4%, *p* = 0.002) and SPA (−3.6 ± 3.0%, *p* = 0.004). For the submandibular glands, DynamicARC techniques (DPA, SPA, and SFA) showed varying differences relative to IMPT. Neither IMPT nor DynamicARC plans met the compliance threshold for these glands. This non‐compliance is primarily attributed to the overlap of target volumes with the submandibular glands, which presents a challenge in sparing these structures while maintaining adequate target coverage.

For D_0.03cc_ in the brachial plexus, brainstem PRV, mandible, spinal cord, and spinal cord PRV, all techniques generally adhered to compliance criteria, except for the ipsilateral brachial plexus. In the ipsilateral brachial plexus, the D_0.03cc_ reduction ranged from −1.0 ± 2.9% to −2.4 ± 2.7% when compared to IMPT. Similarly, for the contralateral brachial plexus, DynamicARC techniques achieved an average D_0.03cc_ reduction of −0.4 ± 2.6% to −1.7 ± 2.3% compared to IMPT. For the brainstem PRV, DynamicARC techniques significantly reduced D_0.03cc_ relative to IMPT and remained well below the compliance threshold. SFA had the greatest reduction (−59.6 ± 3.8%, *p* = 0.004), followed by DPA (−59.1 ± 7.6%, *p* = 0.002) and SPA (−58.1 ± 10.0%, *p* = 0.004). The D_0.03cc_ to the mandible was comparable between IMPT and all three DynamicARC techniques (*p* > 0.05) For the spinal cord and spinal cord PRV, DPA provided the largest reduction in D_0.03cc_ compared to IMPT (−60.0 ± 4.6%, *p* = 0.002), followed by SFA and SPA.

In terms of integral dose, DynamicARC techniques showed varying degrees of reduction compared to IMPT. SPA demonstrated the highest reduction (−5.7 ± 3.0%, *p* = 0.002), followed by DPA (−3.7 ± 3.5%, *p* = 0.025). SFA exhibited minimal change (−0.3 ± 4.8%, *p* = 0.846), with greater variability. For D_1cc_ to the external, with a compliance threshold of ≤73.5%, all techniques met the criteria. In comparison to IMPT, DPA provided the largest decrease (−2.3 ± 1.2%, *p* = 0.002), closely followed by SFA (−2.0 ± 1.5%, *p* = 0.008). SPA, however, exhibited a smaller and non‐significant reduction compared to IMPT (−0.9 ± 1.5%, *p* = 0.148).

### 3.3 Delivery time

A comparison of delivery time across three DynamicARC techniques—DPA, SPA, and SFA—highlights the differences in treatment efficiency. The DPA technique exhibited the highest average delivery time, at 468 ± 46 seconds. DPA produced an average of 214 ± 0 energy layers and 67,179 ± 16,524 spots. SFA had an average delivery time of 427 ± 41 seconds, producing 179 ± 0 energy layers and 66,560 ± 14,984 spots. SPA had the lowest average delivery time, recorded at 246 ± 26 seconds, with a corresponding 107 ± 0 energy layers and 35,147 ± 8,486 spots, indicating it is the most efficient technique in terms of shorter delivery time for fewer energy layers and spots.

## 4 DISCUSSION

The current study evaluated the feasibility and dosimetric advantages of utilizing the partial arc technique in conjunction with the DynamicARC on an IBA ProteusOne PBS machine for treating bilateral HNC. This investigation primarily focused on assessing DynamicARC's adherence to NRG HN009 dosimetric criteria and comparing its efficacy against conventional IMPT. By analyzing how limiting gantry angles influence DynamicARC plan quality, the study offers dosimetric information for proton therapy centers, particularly those equipped with ProteusOne that lack full gantry capabilities.

**FIGURE 5:**
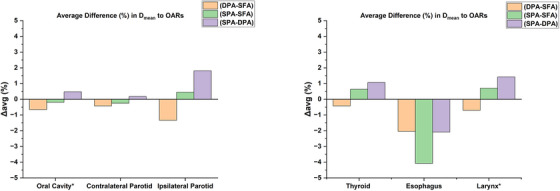
Average cumulative DVHs for oral cavity, larynx, contralateral parotid, and ipsilateral parotid. These DVHs were generated across treatment plans of ten bilateral HNC patients. The plans were generated using three‐field IMPT and DynamicARC PBS proton therapy techniques, including DPA, SPA, and SFA. DVH, dose‐volume histogram; DPA, dual‐partial‐arcs; HNC, head and neck cancer; IMPT, intensity‐modulated proton therapy; PBS, pencil beam scanning; SPA, single‐partial‐arc; SFA, single‐full‐arc.

Both IMPT and DynamicARC plans demonstrated substantial compliance with the NRG HN009 dosimetric criteria across various critical structures, as shown in Figure 4. Comparative analysis between DynamicARC (DPA, SPA, and SFA) and IMPT revealed dosimetric advantages for DynamicARC in sparing the majority of OARs in HNC treatment. [Table 1, Figure 4, Figure 5] The D_mean_ to the parotid glands was lower with all three DynamicARC techniques by an average difference of 14.5%–22.1% compared to IMPT. These findings are consistent with those reported by Liu et al.,^6^ where PAT reduced the D_mean_ to the parotid glands by 20.8%–25.8% compared to IMPT, and by de Jong et al.,^5^ who reported reductions of 20%–23% using PAT compared to IMPT. For the oral cavity, DynamicARC reduced the D_mean_ by an average difference of 15.75% compared to IMPT, aligning closely with reductions of 20.3% reported by Liu et al.^6^ and 13% by de Jong et al.^5^ using PAT techniques. These consistent findings across studies emphasize the potential of arc‐based proton therapy such as DynamicARC, in effectively sparing the parotid glands and oral cavity. Notably, our results indicate that both DPA and SFA techniques achieved similar dose reductions to the parotid glands and oral cavity compared to IMPT. It is important to consider that our IMPT planning utilized a three‐field technique; increasing the number of fields in IMPT could potentially yield different dosimetric outcomes. This variation in beam number may influence the magnitude of dose differences observed between DynamicARC and IMPT plans.

To contextualize our findings with previous studies, we examined the D_1cc_ results for the spinal cord and brain stem. Liu et al.^6^ reported a D_1cc_ to the spinal cord of 17.2 ± 7.0 Gy(RBE) with PAT, compared to 21.7 ± 7.9 Gy(RBE) with IMPT. Similarly, de Jong et al.^5^ reported a lower D_1cc_ to the spinal cord with PAT (7.6 ± 5.8 Gy(RBE)) compared to IMPT (34.5 ± 6.0 Gy(RBE)). Our study demonstrated a reduction in D_1cc_ to the spinal cord with DPA (8.8 ± 0.4 Gy(RBE) compared to IMPT (16.9 ± 3.1 Gy(RBE). Additionally, DPA resulted in a reduction in D_1cc_ to the brainstem compared to IMPT (5.9 ± 1.2 Gy(RBE) vs. 21.9 ± 6.6 Gy(RBE)), consistent with reductions using PAT in studies by Liu et al.^6^ and de Jong et al.^5^

The DynamicARC results from our study demonstrated that restricting gantry angles (e.g., DPA and SPA techniques) compared to full gantry (e.g., SFA technique) did not compromise the overall dosimetric quality. One of the key advantages of DynamicARC therapy is the use of continuous gantry rotation, which allows the optimizer to select from a broader array of beam angles and facilitates more sophisticated dose modulation than static IMPT. This increased flexibility with DynamicARC enables the treatment planner to avoid critical structures more effectively while maintaining high conformality to the target volume. Additionally, the absence of a range shifter in DynamicARC plans results in smaller spot sizes, which effectively reduces the lateral penumbra and minimizes dose spillage to nearby OARs. In contrast, IMPT plans in our study utilized a range shifter, which can lead to an increased air gap between the range shifter and the patient's body. This increased air gap tends to enlarge the spot size,^17^, ^18^ thereby enhancing the lateral penumbra and increasing dose spillage to nearby OARs.

In evaluating low‐dose bath and total integral dose, our study found that the V_3Gy_ of the External was higher for all three DynamicARC techniques than IMPT, whereas previous studies^5^, ^6^ reported lower V_3Gy_ with PAT compared to IMPT. This discrepancy may be attributed to differences in planning and optimization techniques between our study and previous studies. The total integral dose evaluation showed that SFA produced an average reduction of 0.3% relative to IMPT. However, DPA and SPA techniques resulted in an average reduction of 3.7% to 5.7% in total integral dose compared to IMPT. These findings align with Liu et al.^6^ and de Jong et al.,^5^ demonstrating that PAT could result in a lower total integral dose in bilateral HNC treatment compared to IMPT.

**FIGURE 6:**
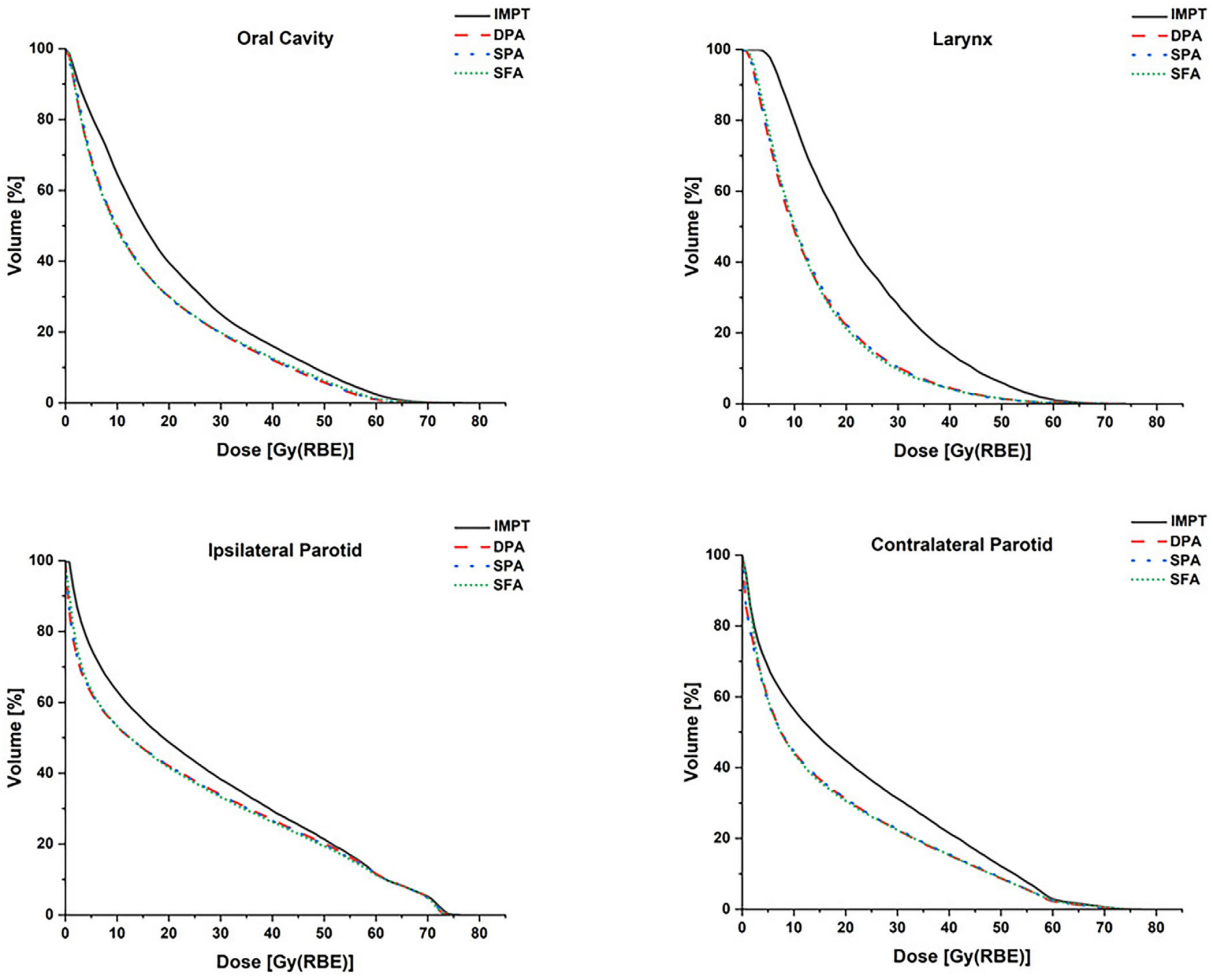
The average difference (%) in D_mean_ to various OARs between DynamicARC techniques. Note: (DPA–SFA) and (SPA–SFA) indicate that the D_mean_ result from the SFA plan was subtracted from the DPA plan and (SPA plan, respectively. Similarly, (SPA–DPA) means the result from the DPA plan was subtracted from the SPA plan. DPA, dual‐partial‐arcs; OARs, organs at risk; SPA, single‐partial‐arc; SFA, single‐full‐arc.

Due to the lack of a 360⁰ gantry system on ProteusOne, the full arc technique is not feasible for clinical treatments in this system. Therefore, the DPA and SPA provide alternative options to treat bilateral HNC patients on ProteusOne. Compared to SPA, DPA produced more conformal distributions and provided slightly better OAR sparing, except in terms of low‐dose bath and total integral dose metrics. Although DPA may offer slightly better dosimetric improvements [Figure 6], one of the key advantages of the SPA is its potential to reduce treatment time. By limiting the number of partial arcs from 2 to 1, the SPA technique's beam delivery time was lowered by 46.9% to 48.2% compared to DPA. This reduction in beam delivery time can be clinically beneficial in high‐throughput environments or for patients who may have difficulty remaining still for extended periods. However, the potential reduction in total treatment time with SPA must be weighed against its slightly lower efficacy in OAR sparing compared to the DPA technique on ProteusOne.

One potential strategy to enhance SPA's dosimetric performance involves increasing the number of energy layers and spots. For instance, in our current DynamicARC planning technique, SPA uses 107 ± 0 energy layers and delivers 35,147 ± 8,486 spots—approximately half the number of both energy layers (214 ± 0) and spots (67,179 ± 16,524) in DPA. Increasing the number of energy layers and spots in SPA plans could improve dose conformality and OAR sparing to levels comparable with DPA. However, this dosimetric improvement would come at the expense of increased delivery time due to additional energy layers switching. These considerations highlight the inherent trade‐offs between treatment time and dosimetric quality, underscoring the importance of selecting the appropriate partial arc technique on ProteusOne based on clinical priorities and patient‐specific needs.

In our study, we employed a couch angle fixed at zero degrees to investigate the feasibility of partial arcs on the ProteusOne system, though non‐coplanar arcs could also be utilized. The use of the DynamicARC technique on ProteusOne with either two coplanar partial arcs or a partial single arc can simplify the treatment setup and reduce the need for complex couch adjustments. The DynamicARC technique with partial arcs with no couch kicks has the potential to streamline the treatment process, thereby improving patient throughput. As partial gantry systems gain traction in proton therapy due to their cost‐effectiveness and reduced operational complexity, our findings highlight that partial arc techniques can deliver dosimetric advantages that are superior to those of IMPT. This approach demonstrates the potential to make DyanmicARC proton therapy more accessible and practical, without compromising treatment quality.

Currently, PBS proton therapy delivery using the DynamicARC technique is not clinical. However, the availability of the DynamicARC treatment planning module in the research version of RayStation allows for testing this planning technique and understanding its dosimetric benefits. The present study was conducted based on the manufacturer's recommended treatment planning guidelines. As experience with DynamicARC planning grows, it is likely that the dosimetric results could be further improved. The full benefits of DynamicARC will be realized once this beam delivery technique is available for clinical use. Future studies should quantify the treatment efficiency of DynamicARC for bilateral HNC in real clinical scenarios. Exploring the practical implementation of DynamicARC with partial gantry systems will be crucial in validating its effectiveness and facilitating its broader adoption in clinical practice.

## 5 CONCLUSION

All four techniques—IMPT, DPA, SPA, and SFA—demonstrated substantial compliance with NRG HN009 dosimetric criteria across critical structures in the treatment of bilateral HNC. On the ProteusOne system, with its 220‐degree gantry range, the use of DPA and SPA techniques provided clear dosimetric advantages over three‐field IMPT by delivering reduced doses to the OARs, achieving a more homogeneous dose within the target volume, and yielding more conformal dose distributions. Future clinical implementation and further research into optimizing DynamicARC protocols are warranted to fully realize its benefits in clinical settings.

## AUTHOR CONTRIBUTIONS

All authors listed contributed to the content of this paper including study design, data collection, data analysis, and writing/editing the manuscript.

## FUNDING INFORMATION

This research did not receive any specific grant from funding agencies in the public, commercial, or not‐for‐profit sectors.

## CONFLICT OF INTEREST STATEMENT

The authors declare no conflicts of interest.
